# Consequences of the perivascular niche remodeling for tumoricidal T-cell trafficking into metastasis of ovarian cancer

**DOI:** 10.21203/rs.3.rs-4940287/v1

**Published:** 2024-09-17

**Authors:** Danuta Kozbor, Marta Winkler, Nemi Malhotra, Anna Mistarz, Sophie Wang, Alan Hutson, Andrea Gambotto, Scott Abrams, Prashant Singh, Song Liu, Kunle Odunsi, Jianmin Wang

**Affiliations:** Roswell Park Cancer Institute; Roswell Park Cancer Institute; Roswell Park Cancer Institute; Roswell Park Cancer Institute; Roswell Park Cancer Institute; Roswell Park Comprehensive Cancer Center; University of Pittsburgh; Roswell Park Comprehensive Cancer Center; Roswell Park Comprehensive Cancer Center; Roswell Park Comprehensive Cancer Center; University of Chicago Medicine Comprehensive Cancer Center; Roswell Park Cancer Institute

## Abstract

The treatment-induced activation level within the perivascular tumor microenvironment (TME) that supports T-cell trafficking and optimal T-cell differentiation is unknown. We investigated the mechanisms by which inflammatory responses generated by tumor-specific T cells delivered to ovarian tumor-bearing mice alone or after oncolytic vaccinia virus-driven immunogenic cancer cell death affect antitumor efficacy. Analyses of the perivascular TME by spatially resolved omics technologies revealed reduced immunosuppression and increased tumoricidal T-cell trafficking and function after moderate inflammatory responses driven by a CXCR4 antagonist-armed oncolytic virus. Neither weak nor high inflammation created a permissive TME for T-cell trafficking. Notably, treatment-mediated differences in T-cell effector programs acquired within the perivascular TME contrasted with comparable antigenic priming in the tumor-draining lymph nodes regardless of the activation mode of antigen-presenting cells. These findings provide new insights into combinatorial treatment strategies that enable tumor-specific T cells to overcome multiple barriers for enhanced trafficking and control of tumor growth.

## Introduction

The limited therapeutic benefit of immune checkpoint blockade (ICB)^1, 2^ in ovarian cancer (OC) patients underscores the need for novel approaches combined with a better understanding of tumoricidal T-cell trafficking across tumor vascular checkpoints^3, 4, 5^. Overexpression of proangiogenic molecules, such as vascular endothelial growth factor (VEGF), results in the formation of immature, tortuous, and hyper-permeable aberrant blood vessels, which are inefficient at nutrient delivery due to impaired blood ow^6, 7^. Additionally, tumor vessels are not only structurally abnormal but also functionally deficient, as T-cell trafficking involves the interplay between T cells and the endothelium, including a well-defined lymphocyte adhesion cascade essential for accessing the tumor microenvironment (TME)^8, 9^. Given the intricate relationship between the tumor vasculature and TME, where blood and lymphatic vessels regulate immune cell access to the tumor and exert direct immunosuppressive effects via endothelial cells and angiogenic factors, combining antiangiogenic therapy and immunotherapy has been explored in various preclinical models and serves as the foundation for several clinical trials^3^. Recently, accumulating preclinical and clinical data suggest that the induction of immunogenic cell death (ICD) in tumor cells is relevant for the efficacy of cancer immunotherapy^10^ as it provides both antigenicity and adjuvanticity required for adaptive immune responses that can be properly executed only in the presence of a permissive TME^11^. However, the magnitude of adjuvanticity within the perivascular TME that supports T-cell trafficking and T-cell differentiation remains incompletely understood.

Accumulated preclinical and clinical data suggest that infection of cancer cells with oncolytic vaccinia virus (OV) stimulates the release of ICD^12^ markers and activation of dendritic cells (DCs)^13, 14^. Our previous findings demonstrated that targeting the VEGF-induced proangiogenic CXCL12/CXCR4 signaling pathway^15, 16^ with an OV expressing a CXCR4 antagonist fused in-frame with the Fc portion of murine IgG (OV-CXCR4-A) effectively inhibited the recruitment of circulating endothelial progenitor cells (CEPs) into the TME in breast and OC-bearing mice^17, 18^ along with inhibition of cancer-associated fibroblasts (CAFs)^19^, myeloid-derived suppressor cells (MDSCs)^20^, and regulatory T (Treg) cells^21^. Here, we investigated the effect of an interplay between the magnitude of inflammatory responses and immunosuppressive network in the perivascular TME on the trafficking of tumor-specific T cells into OC metastasis. T cells expressing a rearranged TCR transgene specific for SV40 T antigen (TCR_TAG_)^22, 23^ were adoptively transferred into TAG^+^ MOVCAR 5009 ovarian tumor-bearing SCID mice alone or following treatment with either OV-CXCR4-A or its control counterpart expressing the Fc portion of murine IgG (OV-Fc). The same antigenicity setting of adoptively transferred T cells within the treatment groups allowed us to focus on the magnitude of inflammation within the perivascular TME required for effector function acquisition by tumor-infiltrating TCR_TAG_ cells. We found that although activation signals delivered in the absence of virally driven ICD led to weak T-cell responses, the high immunostimulation levels generated by the control virus were associated with an increased influx of immunosuppressive myeloid cells and an impediment of antitumor T-cell responses. In contrast, secretion of CXCR4-A into the perivascular tumor areas from virally infected tumor cells led to milder inflammation that modulated the level and quality of myeloid cell responses associated with generating functional TCR_TAG_ cells. The analysis of the perivascular TME by spatially resolved omics technologies revealed treatment-mediated differences in T-cell trafficking in the omental metastasis despite a similar fortification of the tumor vessels by pericyte coverage and comparable expression profiles of adhesion molecules on the tumor vascular endothelium. The treatment-mediated differences in T-cell effector differentiation in the TME, associated with antitumor efficacy, contrasted with comparable antigenic priming of TCR_TAG_ cells in the tumor-draining lymph nodes (tdLNs). These results emphasize the critical role of the level and quality of co-stimulatory signals in the perivascular TME of the OC metastasis on successful effector programming of tumor-infiltrating T cells.

## Results

### OV-CXCR4-A treatment augments the antitumor efficacy of adoptively transferred TCR_TAG_ splenocytes in MOVCAR 5009-challenged SCID mice

**Figure 1:**
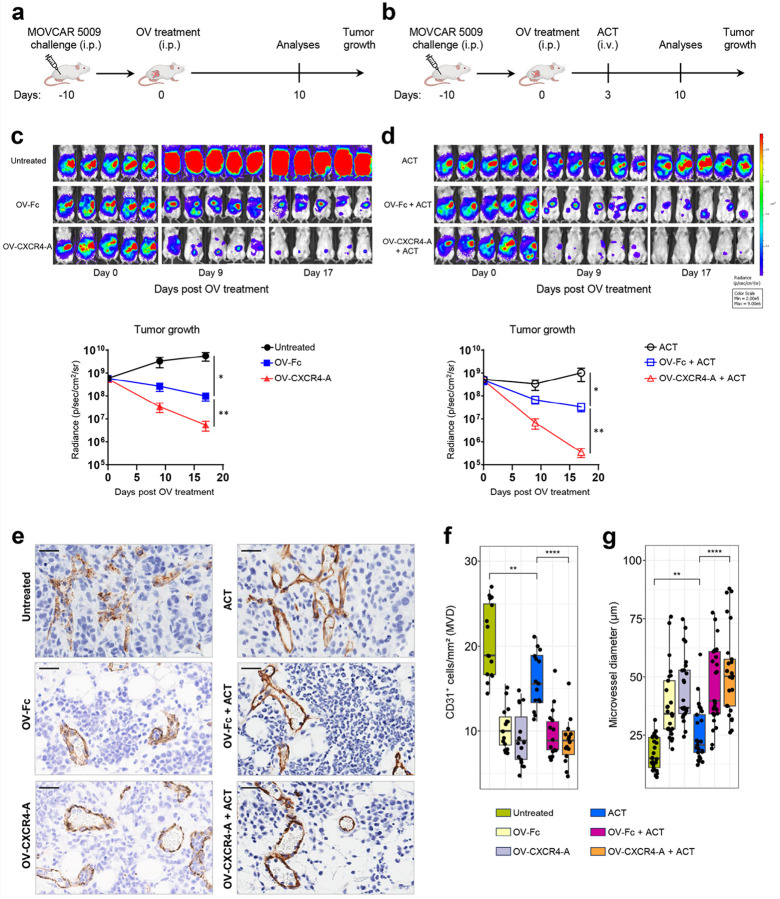
The effect of OV and ACT treatments on progression of MOVCAR 5009 tumor growth and changes in the tumor vasculature. **a, b** Experimental schemes of the treatments. Female SCID mice (*n* = 5 per group) were injected i.p. with 5 × 10^6^ TAG^+^ MOVCAR 5009 cells and treated i.p. with OV-Fc or OV-CXCR4-A 10 days later (**a**). For the combination treatment, mice were injected i.v. with 5 × 10^6^ tumor-specific TCR_TAG_ cells 3 days post-OV treatment (**b**). Control mice were treated with PBS. **c, d** Tumor progression was monitored by bioluminescence imaging on days 0, 9, and 17 post-OV treatment (*upper panels*). Tumor volume curves in SCID mice after different treatments (*lower panel*). The results are representative of three independent experiments with similar results. Individual data points represent mean ± SD. The statistical analysis was performed using two-way ANOVA. *p < 0.05; **p < 0.01. **e** IHC staining of omental tumor sections with CD31-specific mAb. Scale bars: 30 μm. Box plots displaying tumor MVD (n=15/group) (**f**) and microvessel diameter (n=25/group) (**g**). The results are representative of two independent experiments with similar results. *P* values were calculated by a two-sided Wilcoxon rank sum exact test. **p < 0.01; ****p < 0.0001.

To address the antitumor effect of oncolytic virotherapy when delivered alone or in combination with adoptive cell transfer (ACT) of tumor-specific TCR_TAG_ cells, we used a highly metastatic TAG^+^ MOVCAR 5009 ovarian tumor grown orthotopically in SCID mice. As depicted in Fig. 1a, MOVCAR 5009 cells (5 × 10^6^/mouse) were injected intraperitoneally (i.p.) into mice, which were then either left untreated or treated ten days later with OV-Fc or OV-CXCR4-A delivered i.p. at 5 × 10^7^ plaque-forming units (PFU)/mouse. TCR_TAG_ splenocytes isolated from transgenic B6.Cg-Tg(TcraY1,TcrbY1)416Tev/J mice were injected intravenously (i.v.) into tumor-bearing mice (5 × 10^6^ cells/mouse) three days after OV treatment (Fig. 1b). This timing was chosen to exploit the effect of virally induced ICD on T-cell trafficking and activation while avoiding decreased vessel perfusion that occurs within the first 48 hours of OV delivery and subsequent revascularization that starts ten days later^24^. Inhibition of tumor growth, quantified by bioluminescence imaging (Fig. 1c and d *upper panels*), revealed that although both viruses significantly inhibited tumor growth compared to untreated tumor-bearing mice, treatment with OV-CXCR4-A exhibited higher efficacy compared to the control counterpart (Fig. 1c, *lower panel*; *p* < 0.01). This could be due to inhibition of the immunosuppressive network by the CXCR4-A protein released from infected tumor cells as well as direct antitumor activity of CXCR4-A through induction of apoptosis, ADCC, and CDC^17, 18, 25^. A short-lasting inhibition of tumor growth was observed following the ACT monotherapy, indicating the antitumor activities of TCR_TAG_ cells. The OV/ACT combination treatments increased antitumor efficacies, though the effect was more profound in tumor-bearing mice treated with OV-CXCR4-A than OV-Fc (Fig. 1d, *lower panel*; *p* < 0.01).

#### Restoration of aberrant tumor blood vessels after OV/ACT combination treatments

**Figure 2:**
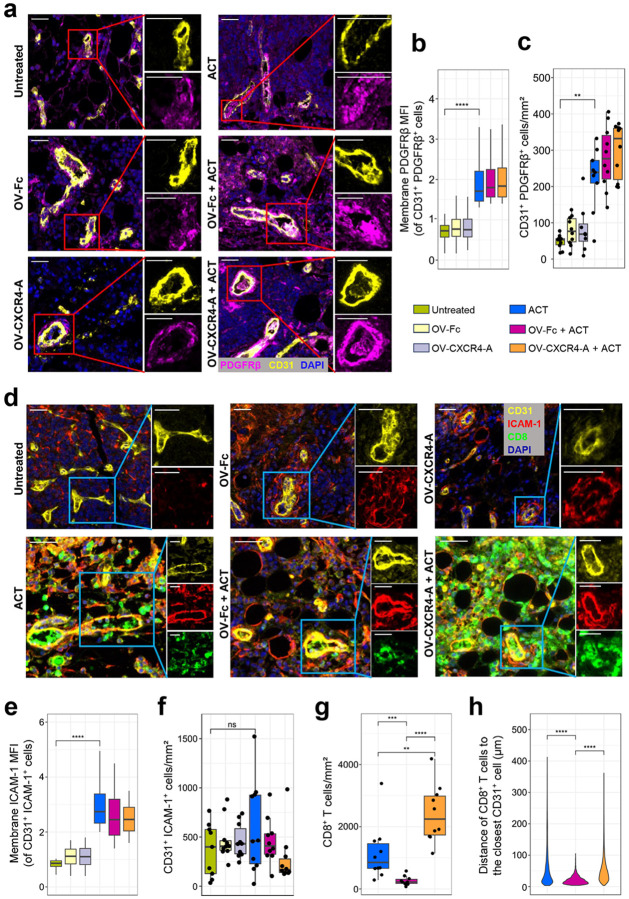
Restoration of aberrant tumor blood vessels after OV and ACT treatments. **a** Representative mIF images showing treatment-mediated changes in the expression of CD31 and PDGFRβ on the tumor vasculature of MOVCAR 5009 omental tumors. The nuclei were visualized by DAPI staining. CD31-Opal 570, PDGFRβ-Opal 520. Scale bars: 30 μm. **b** Box plots of PDGFRβ expression levels on the tumor vasculature (n=8–10/group). **c** MVD of CD31^+^PDGFRβ^+^ cells in omental tumors among different groups of tumor-bearing mice (n=8–10/group). **d** Representative mIF images showing changes in the expression of CD31 and ICAM-1 on the tumor vascular ECs (*upper panel*), and CD8 on adoptively transferred CD8^+^ TCR_TAG_ (*lower panel*). CD31-Opal 570, ICAM-1-Opal 620, CD8-Opal 690. Scale bars: 30 μm. **e** Boxplots of ICAM-1 expression on CD31^+^ ECs (n=9–10/group). **f** Boxplots displaying the MVD of CD31^+^ICAM-1^+^ ECs (n=9–10/group). **g** Accumulation of CD8^+^ cells in the perivascular area in omental tumor metastasis (n=10/group). **h** Co-localization of CD8^+^ T cells relative to the closest CD31^+^ vascular ECs (n=10/group). *P* values were calculated by a two-sided Wilcoxon rank sum exact test. **p < 0.01; ***p < 0.001; ****p < 0.0001; ns – non significant.

As the abnormal tumor vasculature affects the infiltration of immune cells and indirectly promotes immune suppression through the synthesis of proangiogenic receptors and factors^26^, we examined changes in microvessel density (MVD) and size in the omental tumors ten days after treatment initiation. Immunohistochemical staining (IHC) of omental tumor sections from untreated MOVCAR 5009-bearing mice with anti-PECAM-1/CD31 mAb revealed a chaotic blood vessel network with high MVD and small luminal diameters compared to the OV-treatment groups (Fig. 1e, *left panel*). Virotherapy treatment with either OV-Fc or OV-CXCR4-A reduced the MVD by approximately twofold compared to the untreated tumor along with increased luminal diameters (Fig. 1f and g), supporting the previously reported pruning ability of OVs by infecting proliferating tumor vascular ECs^24, 27^. Consistent with the findings of IFNγ-mediated normalization of tumor vessels by T cells^28, 29^, the MVD decreased along with increases in luminal diameters after adoptively transferred TCR_TAG_ cells, though the effect was less prominent than that mediated by OV treatment (Fig. 1e *right panel*; f and g). We next examined the fortification of the blood vessels by pericyte coverage that provides structural support while filling the gap between ECs^30^, the lack of which constitutes one of the inhibitory mechanisms limiting T-cell transendothelial migration^31^. Paraffin sections of omental tumors from all treatment groups were analyzed for expression of CD31 and platelet-derived growth factor receptor b (PDGFRb) on the tumor vasculature using multispectral immunofluorescence (mIF) imaging after staining with specific mAbs. Figure 2a (*left panel*) shows that most of the blood vessels in untreated tumors or after treatment with either OV-Fc or OV-CXCR4-A were poorly covered by PDGFRb-expressing pericytes (Fig. 2b and c), indicating that virotherapy treatment alone does not contribute to the vascular normalization process. On the other hand, the increased levels of PDGFRb expression and density of CD31^+^ and PDGFRb^+^ cells after treatment with ACT alone or in combination with OV (Fig. 2a, *right panel*), pointed to an improved restoration of aberrant tumor vasculature (Fig. 2b and c). This was accompanied by increased expression of intercellular adhesion molecule ICAM-1 (CD54) that interacts with integrin molecules on T cells during their extravasation to the tumor bed^32^. The ICAM-1 levels on CD31-expressing ECs in untreated or virotherapy-treated tumors (Fig. 2d, *upper panel*) were lower compared to those receiving ACT alone or in combination with either OV-Fc or OV-CXCR4-A (Fig. 2d, *lower panel*, and e), though the numbers of CD31^+^ICAM-1^+^ cells were similar in all treatment groups (Fig. 2f). Despite the comparable expression of ICAM-1, the trafficking of CD8^+^ TCR_TAG_ cells into the tumors differed (Fig. 2d, *lower panel*). As shown in Fig. 2g, the lowest accumulation of CD8^+^TCR_TAG_ cells in the perivascular tumor areas was detected after OV-Fc treatment (264.3 ± 46.1 cells / mm^2^) compared to approximately 5- and 10-fold higher numbers in the untreated and OV-CXCR4-A-treated tumors (1,150.8 ± 285.4 cells / mm^2^ and 2,417.6 ± 291.1 cells / mm^2^, respectively). While T cells after OV-Fc treatment were concentrated mostly within the 50 μm area surrounding the blood vessels, they migrated within the perivascular areas of untreated and OV-CXCR4-A-treated tumors (Fig. 2h).

### Transcriptomic profiles of endothelial adhesion molecules (EAMs), angiogenic receptors, and growth factors after ACT and OV treatments

**Figure 3:**
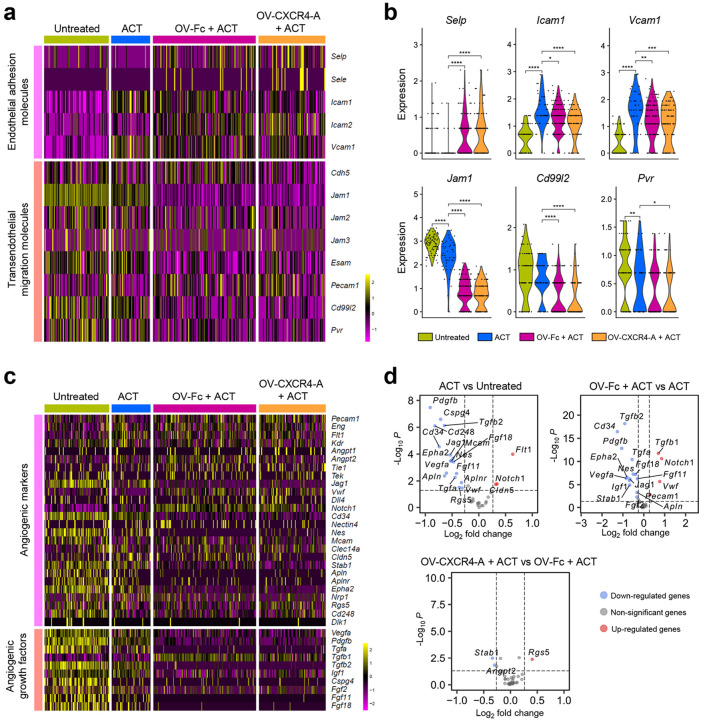
Changes in transcriptomic profiles of EAMs and angiogenic markers after ACT and OV treatments. **a** Heatmap displaying the expression of genes encoding EAMs and transendothelial migration molecules on the tumor vascular endothelium. **b** Violin plots comparing the expression levels of genes whose expression differs significantly among the treatment groups. Pvalues were calculated by a two-sided Wilcoxon rank sum exact test. *p< 0.05; **p < 0.01; ***p < 0.001; ****p < 0.0001. **c** Heatmap displaying the expression of the genes encoding angiogenic markers and growth factors in the perivascular TME of omental metastasis. **d** Volcano plots demonstrating enrichment of differentially expressed markers of angiogenesis between the treatment groups. Each blue and red dot denotes an individual gene with a Benjamini-Hochberg-adjusted *p* < 0.05 and fold change > 1.2.

We next performed spatial transcriptomic (ST) analyses of the perivascular TME using omental tumor sections isolated ten days after treatment initiation to thoroughly address the complexity of T-cell trafficking through the vascular barrier^32^. Five to seven regions of interest (ROIs) on formalin-fixed paraffin-embedded (FFPE) tissues with well-organized CD31-expressing tumor vasculature networks were identified by a pathologist within each omental tumor section (Supplementary Fig. 1a). Within each ROI, 55 × 55 μm spots in size that accumulated no more than 10 cells were selected based on the expression of genes encoding *Pecam1* and at least one *Cd3d* transcript and used for the transcriptomic analysis of the perivascular TME. As shown in Fig. 3a and b, the transcription levels of endothelial E/P selectin genes (*Sele* and *Selp*) were low in all analyzed tumor sections with *Selp* increasing only after combination treatments. Consistent with the mIF imaging, *Icam1* expression was upregulated in all treatment groups along with vascular cell adhesion molecule 1 (*Vcam1*). Surprisingly, the transcriptomic profiles of EC adhesion molecules involved in transendothelial migration (TEM)/diapedesis processes^32^, including junctional adhesion molecules (*Jam1/F11r*), *Cd99l2*, and *Pvr* were higher in the untreated and TCR_TAG_-treated tumors than those receiving the combination-treatments. These differences could reflect treatment-mediated changes in the dominant cell types surrounding the perivascular TME. We also detected decreased expression of genes promoting angiogenesis (*Cd34, Jag1, Epha2, Cd248, Nes, Apln, and Aplnr*) and angiogenic growth factors (*Vegfa, Pdgfb, Tgfb2, and Cspg4*)^33^ after adoptively transferred TCR_TAG_ cells (Fig. 3c and d). This angiogenic effect was further impaired after the OV-Fc treatment combination, except for genes encoding *Vwf*^34^, *Notch1*^35^, and *Tgfb1*^36^, and upregulated transcription of the regulator of the G-protein gene (*Rgs5*)^37^ by OV-CXCR4-A.

#### Treatment-mediated changes in cancer and myeloid cell compositions in the perivascular TME

**Figure 4:**
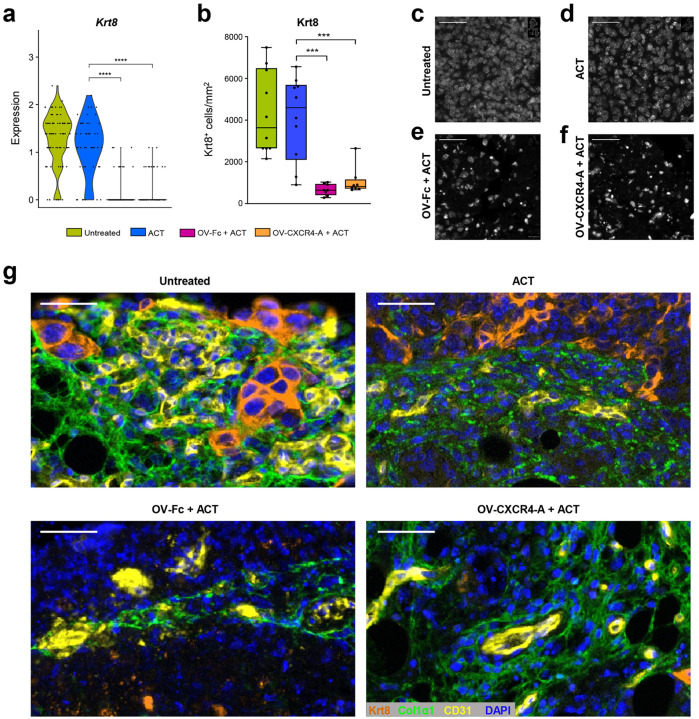
Treatment-mediated changes in keratin 8 expression on MOVCAR 5009 cancer cells and Col1 1 fibers organization in perivascular sections of omental tumors. **a, b** Violin plots of *Krt8* gene expression (**a**) and boxplots of the density of Krt8-positive cancer cells (n=8–10/group) (**b**) in perivascular sections of the omental tumors analyzed by Spatial Transcriptomics and mIF imaging, respectively. *P* values were calculated by a two-sided Wilcoxon rank sum exact test. ***p < 0.001; ****p < 0.0001. **c-f** Representative mIF images depicting tumor cells’ nuclei distribution stained by DAPI. Scale bars: 40 μm. **g** Representative panoramic view illustrating the treatment-mediated changes in colocalization of CD31-expressing vascular ECs, Krt8-expressing tumor cells, and Col1 1 fibers in the perivascular TME of the omental tumors analyzed by mFI imaging. DAPI was applied to visualize the nuclei. CD31-Opal 480, Krt8-Opal 570, Col1 1-Opal 620. Scale bars: 40 μm.

The ST analysis revealed differences in global gene expression levels in the perivascular TME among the treatment groups characterized by low levels of activation generated by the ACT monotherapy, which contrasted with high numbers of activated genes after OV/ACT combined treatments (Supplementary Fig. 1b and c). This could be due to the OV-driven ICD-generated inflammation characterized by the expression of genes encoding danger signals including annexin (*Anxa1*), calreticulin (*Calr*), high mobility group box 1 (*Hmgb1*), heat shock protein alpha (*Hsp90aa1*), and IL-1β (*Il1b*) (Supplementary Fig. 1d). Based on the treatment-induced activation score within the selected ROIs, the highest number of genes with upregulated overall transcription levels were present after the OV-Fc/ACT combination. Among them, 22% of the transcripts were shared by all treatment groups and only 36% of transcripts were shared by the combination with OV-CXCR4-A (Supplementary Fig. 1b). The perivascular TME after the OV-CXCR4-A/ACT treatment had the highest numbers of downregulated genes with 71% overlap with the OV-Fc/ACT group (Supplementary Fig. 1c), indicating a profound modulatory effect of the CXCR4-A protein on cellular composition and/or signaling within the perivascular TME. Among the cellular components of the perivascular TME, analyzed by ST and mIF imaging, cancer cells dominated the landscape of the omental metastasis in untreated mice and those receiving monotherapy with TCR_TAG_ cells, evidenced by higher levels of keratin 8 (*Krt8*) gene expression (Fig. 4a) and density of Krt8^+^ cells (Fig. 4b) with tight concentrations of the tumor cell nuclei (Fig. 4c and d). In contrast, the tumor cell numbers decreased after the combination therapies (Fig. 4a and b), also evidenced by their scattered nuclei (Fig. 4e and f). Analyses of omental tumor sections by mIF imaging revealed that clusters of Krt8^+^ tumor cells were closely adjacent to the tumor vasculature in untreated mice (Fig. 4g). They were gradually relaxed after ACT monotherapy and formed small clusters after the combination treatment with OV-Fc or OV-CXCR4-A (Fig. 4g). Differences were also observed in colocalization of tumor vasculature and Krt8^+^ cells relative to collagen 1 1 (Col1 1) protein fibers that are known for adverse effect on T cell trafficking^38^. In untreated tumors, tightly organized and parallel fibers of Col1 1 were found around the blood vessels or at the tumor-stroma interface, which contrasted with loose networks in the treatment groups (Fig. 4g).

**Figure 5:**
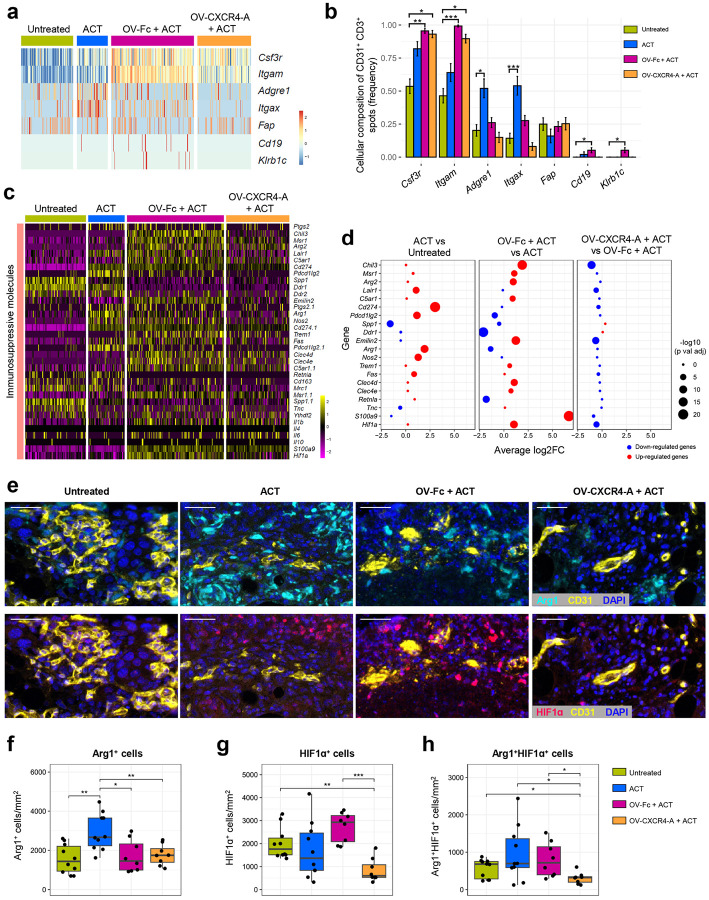
Differences in the influx of myeloid cells and expression of immunosuppressive molecules in the perivascular TME after ACT and OV/ACT combination treatments. **a** Heatmap displaying the row-scaled expression of selected genes linked to different cell populations infiltrating the perivascular TME in omental tumor sections analyzed by spatial transcriptomics. Myeloid cell markers: CSF3R (Csf3r); Cd11b (Itgam); F4/80 (Adgre1): CD11c (Itgax); CAF marker FAP (Fap); B cell marker (Cd19); and NK cell marker NK1.1 (Klrb1c). **b** Frequency of the cell composition in the perivascular niches. Data are presented as mean ± SD, combined with a Fisher Exact Test. * *p* < 0.05; ** *p* < 0.01; ***p < 0.001. **c** Heatmap of normalized gene expression associated with immunosuppressive myeloid cell subsets. **d** Dot plot presentation of differences in immunosuppressive gene expression among treatment groups. The enhanced expression (red) and reduced expression (blue) dots denote individual genes and the size of the dot shows the negative log10 transferred Benjamini-Hochberg-adjusted *p* values. **e** The representative mIF images of the treatment-mediated changes in colocalization of CD31^+^ vascular ECs and Arg1^+^ cells (upper panel) and HIF1a^+^ cells (*lower panel*). The omental tumor sections were stained with anti-CD31, -Arg1, and-HIF1 mAbs, counterstained with DAPI to visualize nuclei, and analyzed on the PhenoImager HT. CD31-Opal 480, Arg1-Opal 690, HIF1 -Opal 520. The same ROIs are presented as in Fig. 4g. Scale bars: 40 μm. **f** Box plots show the number of Arg1^+^ cells, (**g**) HIF1a^+^ cells, and (**h**) double-positive Arg1^+^HIF1a^+^ cells within the analyzed ROIs (n=8–10/group). The results are representative of two independent experiments with similar results. *P* values were calculated by a two-sided Wilcoxon rank sum exact test. *p < 0.5; **p < 0.1; ***p < 0.001.

The ST analyses of the inflammation-vascular axis that focused on the cellular components infiltrating the vascular TME revealed the highest accumulation of colony-stimulating factor 3 receptor (*Csf3r*) and CD11b (*Itgam*) expressing neutrophils after the OV/ACT combination therapies, compared to tumors treated with ACT monotherapy or the untreated controls (Fig. 5a and b). Infiltration of F4/80^+^ tumor-associated macrophages (TAMs), expressing *Adgre1*, and CD11c^+^ monocytes/DCs, expressing *Itgax*, were more abundant after monotherapy with TCR_TAG_ cells, whereas frequencies of cancer-associated fibroblasts (CAFs) expressing *Fap*, B cells expressing *Cd19*, and NK cells expressing *Klrb1c* were comparable across all analyzed groups. Consistent with an influx of neutrophils/polymorphonuclear (PMN)-MDSCs into the perivascular area, the expression levels of chemokine receptors and chemokines including *Cxcr2/4*, *Ccr2*, and *Ccl2* dominated the landscape after the OV-Fc/ACT combination though their expression was decreased in animals treated with OV-CXCR4-A instead (Supplementary Fig. 2a and b). The increased transcription of *Ccr5*, along with *Cxcl9/10* and *Ccl5* chemokines after the ACT monotherapy supported higher infiltration of monocytes/DCs and TAMs. Aligned with the accumulating evidence that PMN-MDSCs and M2-like TAMs are key determinants in establishing a T cell-excluded tumor phenotype^39, 40,41, 42^, the transcription of immunosuppressive markers after the ACT monotherapy and OV-Fc/ACT combination treatment was higher (Fig. 5c and d). This included the leukocyte-associated immunoglobulin-like receptor gene (*Lair1*), which inhibits immune cell activation upon binding to collagen and collagen domain-containing proteins as ligands^43^, along with *Cd274* and *Pdcd1lg2* as well as arginase 1 (*Arg1*), nitric oxide synthase (*Nos2*), and Fas. The OV-Fc/ACT combination further augmented the immunosuppression landscape of the perivascular stroma by PMN-MDSC-expressing *Chil3, Msr1, Arg2, C5ar1, Emilin2, Hif1a, Trem1, Clec4d, Clec4e* genes or elevated *S100a9* by mononuclear (M)-MDSCs^44^, though the transcription levels of most of the immunosuppressive molecules were downregulated by the OV-CXCR4-A/ACT treatment combination (Fig. 5c and d). Among the array of immunosuppressive genes whose expression was specifically affected by different treatments were *Arg1*, which was highly expressed after ACT monotherapy, and *Hif1a*, which dominated after OV-Fc/ACT combination. Based on this observation, we examined the expression profile of Arg1 and HIF1a by mIF imaging in the same ROIs employed for the analysis of the colocalization of Krt8^+^ cancer cells, CD31^+^ vasculature, and Col1 1^+^ protein fibers. Consistent with the ST analysis, the highest number of Arg1-expressing tumor-infiltrating myeloid cells was detected after the ACT monotherapy compared to other treatments (Fig. 5e *upper panel*; and f), whereas the expression of Hif1a dominated in myeloid cells after the OV-Fc/ACT combination treatment, with some cells expressing both antigens (Fig. 5e *lower panel*; and g and h, respectively). In contrast, the numbers of cells expressing Hif1a alone or together with Arg1 were reduced almost to background levels in the OV-CXCR4-A/ACT-treated tumors (Fig. 5g and h).

#### The extracellular matrix (ECM) remodeling within the perivascular TME after ACT and OV treatments

**Figure 6:**
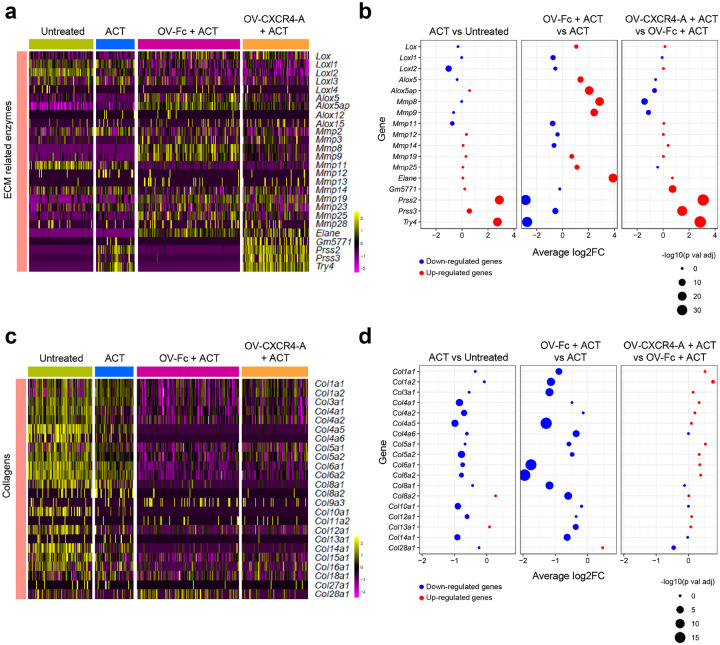
Changes in the gene expression profile of ECM-modulating enzymes and collagen genes in the perivascular TME of omental tumors after ACT and OV treatments. **a** Heatmap showing the expression profile of ECM-modulating enzymes. **b** Dot plot denoting differences in the ECM-modulating enzyme expression levels among the treatment groups. **c** Heatmap showing the expression profile of genes encoding collagens. **d** Dot plot denoting differences in collagen gene expression among treatment groups. The size of the dot denotes a negative log10 adjusted *p* value, and the color shows the up (red) or down (blue) regulation of expression.

The infiltration of different subsets of leukocytes into the perivascular TME was also associated with changes within the ECM composition^45^. The ST analysis revealed that the high accumulation of myeloid cells in OV-Fc-treated omental tumors was associated with increased transcription of some lysyl oxidases (LOX) compared to the other groups of mice (Fig. 6a and b). This included *Lox, Alox5, Alox5ap*, and *Alox15* known for promoting metastasis through involvement in ECM crosslinking and remodeling^46^. Among other soluble factors involved in neutrophil/TAM-mediated ECM remodeling, and whose expression was also elevated in ACT/OV-Fc-treated tumors, were matrix metalloproteinases (MMPs), including *Mmp8, Mmp9, Mmp19, Mmp25*, and neutrophile elastase (*Elane*) genes. This profile of MMP gene expression differed from elevated transcription levels of serine-based endopeptidase and proteases, including *Prss2, Prss3, Gm5771*, and *Try4* after treatment with the ACT monotherapy or its combination with OV-CXCR4-A, highlighting dissimilarities in tumor-infiltrating myeloid cell subsets between OV-Fc and OV-CXCR4-A-treatment groups. Changes in the gene expression profile of ECM-modulating enzymes were also associated with differences in collagen gene expression. The highest expression of genes encoding *Col1a1-2, Col3a1, Col4a1, Col4a2, Col5a1, Col5a2, Col6a1, Col8a1, Col9a3, Col10a1, Col12a1, Col14a1, Col15a1, Col16a1, Col18a1*, and *Col28a1* were detected in untreated tumors (Fig. 6c and d), whose landscapes were dominated by Krt8^+^ cancer cells. The expression levels of *Col4a1/2/5, Col5a2, Col10a1*, and *Col14a1* were lower after the ACT monotherapy and were further downregulated after the OV-Fc/ACT combination treatment, with small changes introduced by OV-CXCR4-A instead of OV-Fc. The transcriptomic changes in the perivascular TME introduced by the OV-CXCR4-A/ACT treatment were characterized by increases in pathways associated with collagen degradation and ECM receptor interaction along with downregulation of apoptosis and hypoxia (Supplementary Fig. 3).

### Differences in effector program acquisition of TCR_TAG_ cells within the perivascular TME contrast with similar T-cell activation profiles in the tdLNs

**Figure 7:**
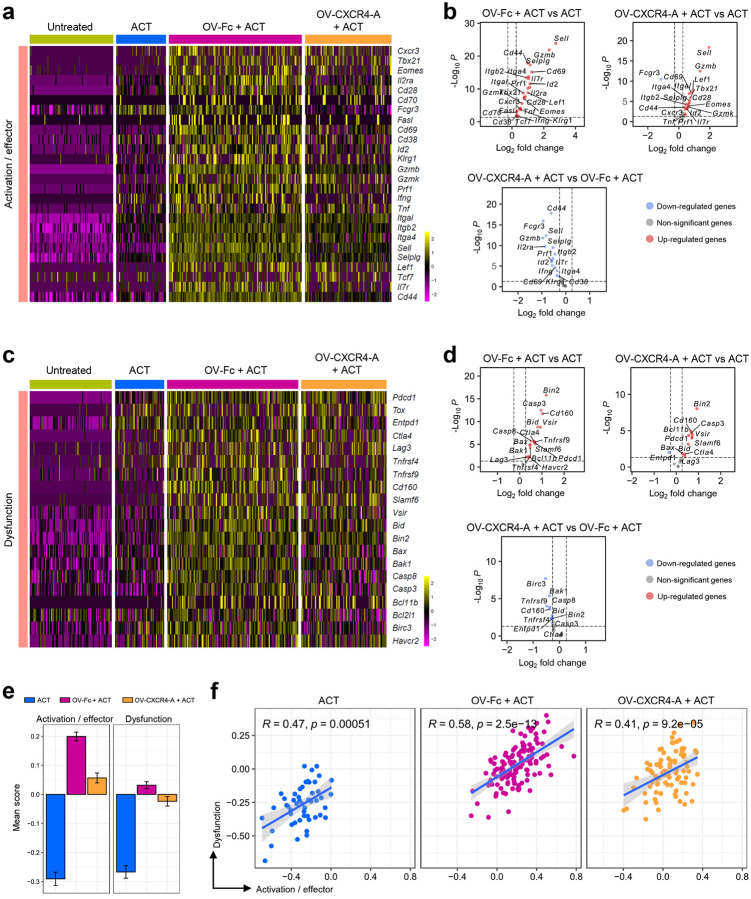
Treatment-mediated differences in effector program acquisition of TCR_TAG_ cells within the perivascular TME. **a-d** The ST analysis of gene expression in TCR_TAG_ cells infiltrating into the perivascular TME of the omental metastasis after ACT and OV treatments. **a** Heatmap of gene expression denoting an activated/effector phenotype of TCR_TAG_ cells. **b** Volcano plots presenting enrichment of differentially expressed genes denoting an activated/effector phenotype of TCR_TAG_ cells among the treatment groups. **c** Heatmap of the expression of genes associated with a dysfunctional phenotype of TCR_TAG_ cells infiltrating the omental tumors. **d** Volcano plots showing enrichment of differentially expressed genes denoting a dysfunctional phenotype of TCR_TAG_ cells among the treatment groups. For the volcano plots, each blue and red dot denotes an individual gene with a Benjamini-Hochberg-adjusted *p* < 0.05. **e** Column graphs presenting treatment-mediated changes in the expression of genes linked to activated/effector and dysfunctional states of tumor-infiltrating TCR_TAG_ cells. Data are presented as mean ± SD of the mean score. **f** Correlations between expression levels of genes associated with activated/effector and dysfunction of TCR_TAG_ cells.

We next examined how the treatment-mediated changes in the perivascular TME affect the activation profile of tumor-infiltrating TCR_TAG_ cells. Figure 7a shows that T cells transferred to untreated tumor-bearing mice exhibited a low expression level of genes associated with stem-like (*Tcf7* and *Il7r*) and activation/effector (*Cd44, Cd69, Cxcr3, Prf1, Gzmb, Sell*) phenotypes, with only small increases in transcription of *Fcgr3, Ifng*, and *Cd38*. This contrasted with high activation profiles of TCR_TAG_ cells induced by the OV/ACT combination treatments with either OV-Fc or OV-CXCR4-A (Fig. 7a and b), in line with the previous findings that co-stimulation signals are required for T cells to differentiate into effector cells^47^. However, the immunostimulatory responses induced by the ACT/OV-Fc treatment contributed to higher transcription of genes associated with activation and effector differentiation of TCR_TAG_ cells compared to those generated by its CXCR4-A-armed counterpart (Fig. 7a and b). These changes appeared more quantitative than qualitative based on the increased transcription of a similar set of genes in both groups compared to the ACT treatment alone (Fig. 7b). Furthermore, the weak activation of TCR_TAG_ cells after the ACT monotherapy treatment was concomitant with reduced expression of genes associated with dysfunction (Fig. 7c), supporting the notion that the lack of co-stimulatory signals generally results in T-cell anergy linked to peripheral tolerance^48^. Some of these genes, including *Pdcd1, Ctla4, Tnfrsf9, Cd160, Slamf6, Vsir, Bid, Bax, Bak1, Casp3*, and *Casp8* were highly expressed by TCR_TAG_ cells after OV-Fc/ACT treatment and to a lesser extent in OV-CXCR4-A/ACT-treated mice (Fig. 7c and d). These differences were also reflected in the mean scores of the overall T-cell responses, being smallest after ACT monotherapy, highest and intermediate after OV-Fc/ACT and OV-CXCR4-A/ACT combinations, respectively (Fig. 7e). Furthermore, correlations between the expression of genes encoding activation/effector and dysfunction markers in TCR_TAG_ cells were observed in all treatment groups (Fig. 7f), with the strongest association in OV-Fc/ACT-treated tumor-bearing mice characterized by impaired T-cell trafficking and reduced antitumor efficacy.

**Figure 8:**
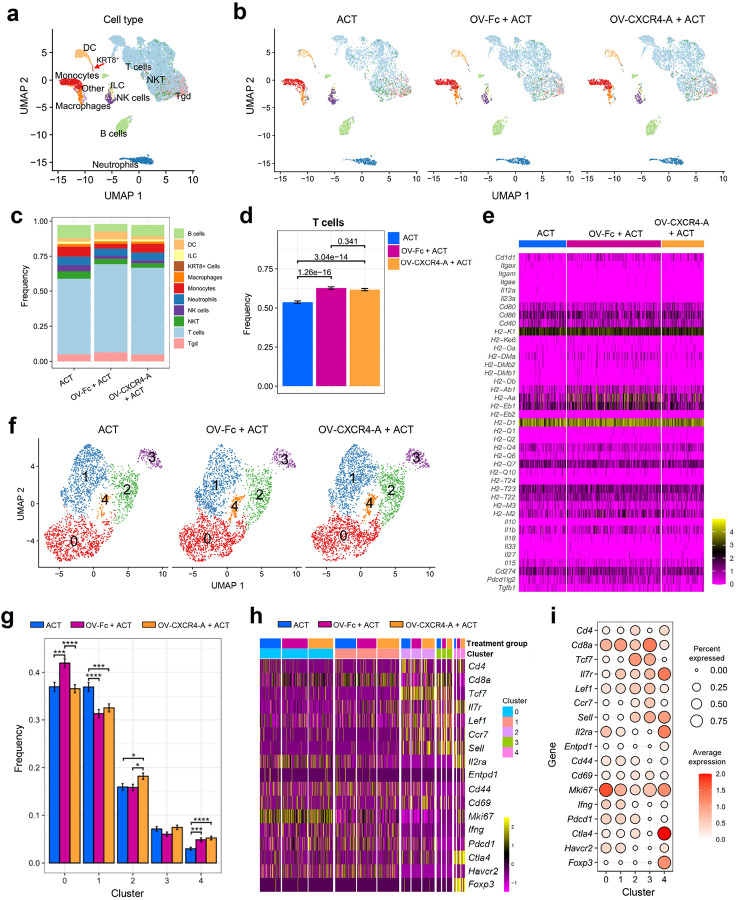
scRNAseq analysis of TCR_TAG_ cells in the tdLNs of MOVCAR 5009-bearing SCID mice after ACT and OV treatments. **a** Uniform manifold approximation and projection (UMAP) visualization of the cell populations in tdLNs analyzed 10 days after OV treatment. The red arrow indicates a population of *Krt8*-expressing tumor cells. **b** UMAP visualization of distinct cell populations in tdLNs from individual treatment groups. Cell type colors correspond to the respective cell type populations. **c** Bar plot representing the frequency of cell type populations in tdLNs after different treatments. Cell types in the data panel are highlighted by matching colors on UMAP plots. **d** Frequency of TCR_TAG_ cell composition with significant changes between the indicated groups. Data are presented as mean ± SD, combined with a Chisquared test. **e** Heatmap of the expression of DC activation markers. **f** UMAP visualization of reclustered TCR_TAG_ cell population comprising five (0–4) T cell clusters in the tdLNs of SCID mice. **g** Bar plot depicting frequencies of each T cell cluster in tdLNs of different treatment groups. Data are presented as mean ± SD, combined with a Chisquared test. * *p* < 0.05; ***p < 0.001, and ****p < 0.0001. **h** Heatmap presenting the expression of selected genes of individual clusters in different treatment groups. **i** Dot plot illustrating the average expression and prevalence of genes among T cell clusters.

To address the potential molecular and cellular crosstalk between the tumor and the lymphoid tissues^49, 50, 51^, we next investigated whether the treatment-mediated changes in the perivascular TME were associated with differences in T-cell priming in the tdLNs. In particular, we focused on antigenic priming of TCR_TAG_ cells by DCs activated in adjuvant settings driven by immune responses that do not rely on ICD versus OV-driven activation. Enlarged inguinal LNs, visible only in the treated tumor-bearing mice, were resected at the time of tumor analysis and subjected to single-cell RNA sequencing (scRNAseq). Figure 8a–d shows similar transcriptomic profiles of individual LN clusters in all treatment groups dominated by T cells, with less than 10% increases after OV/ACT combinations compared to ACT monotherapy. In all treatment groups, less than 0.1% of cells in the tdLNs expressed the Krt8 gene indicating an early onset of metastatic disease. The number of DCs was approximately 2-fold higher after the combined OV-Fc/ACT treatment (Supplementary Fig. 4a, p < 0.0001), which aligns with the highest activation level of tumor-infiltrating TCR_TAG_ cells. However, despite these quantitative differences and some increases in the expression of genes encoding class II antigens *H2-Aa, H2-Ab1, H2-Eb1*, and programmed cell death-ligand 2 (*Pdcd1lg2*) (Supplementary Fig. 4b), the transcriptional profile of key activation antigens was similar (Fig. 8e). This was further reflected in the lack of heterogeneity within the T-cell subsets identified through the cluster analysis (Fig. 8f). T cells within the dominant clusters 0 and 1 were present in increased numbers after OV-Fc/ACT and ACT treatments, respectively (Fig. 8g). They expressed genes associated with activation (*Il2ra, CD44, Cd69, Cd28*) and proliferation (*Mki67*), with low expression of the co-inhibitory receptor *Havcr2*, and were negative for the expression of *Entpd1* associated with exhaustion^52^ (Fig. 8h and i, Supplementary Fig. 5). The majority of T cells in cluster 2, present in an increased number after OV-CXCR4-A/ACT combination, were stem-like (*Tcf7*^+^, *Pdcd1*^+^, and *Havcr2*^*−*^) with the ability to migrate to the tumor site where they undergo differentiation to the effector state^47^, whereas naïve cells (*Sell*^+^ and *Pdcd1*^*−*^) primarily constituted cluster 3. Small numbers of CD4^+^ and CD8^+^ cells expressing *Foxp3, Il2ra*, and *Ctla4*, were concentrated in cluster 4 with increased numbers after the OV/ACT treatments. Consistent with the transcriptomic study, flow cytometry analysis of TCR_TAG_ cells isolated from the tdLNs at the time of scRNAseq revealed a similar profile of CD62L, CD44, CD69, and PD1 expression in all treatment groups (Supplementary Fig. 6a-d). Altogether, the presence of antigen-experienced T cells in the tdLNs of all treatment groups with a stem-like phenotype and the ability to migrate into the tumor bed^47^ suggested the TME as a major relevant site for such alterations in effector T-cell differentiation.

## Discussion

A critical obstacle in advancing immunotherapy as a successful approach to combat OC relies on the multiple pathways of immunosuppression that enable tumor escape. Emerging data have identified the perivascular TME as home to a multitude of cell types engaged in various signaling networks that collectively foster a supportive environment for OC metastasis. Characterizing the signaling pathways and intercellular communication between resident cell types in the perivascular niche is critical to identifying potential OC-specific therapeutic targets. In this study, we combined the OV-based treatment approach with adoptively transferred TCR_TAG_ cells to target TAG^+^MOVCAR 5009 ovarian tumor aiming at a better understanding of the role of the vascular barrier and inflammatory responses in the perivascular TME in regulating T-cell trafficking into omental metastases. We demonstrated that treatment with either the control or CXCR4-A-armed oncolytic vaccinia virus reduced MVD but had no significant effect on PDGFRb and ICAM-1 expression unless used in combination with adoptively transferred TCR_TAG_ cells.

This finding aligns with reports that tumor vascular normalization is linked to increased production of IFN-g by T cells^28, 29^, which reduces endothelial cell VEGF production and stimulates pericyte recruitment^53^. However, despite similar coverage of tumor vessels by pericytes and increased ICAM-1 expression on tumor vascular endothelium, TCR_TAG_ cell trafficking to the tumor bed differed among treated mice and was largely determined by the state of T-cell activation associated with heterogeneity of tumor-infiltrating myeloid cells and ECM remodeling. These findings highlight the pivotal role of magnitude and quality of co-stimulatory signals in the perivascular niche received by tumor-specific T cells in regulating T-cell differentiation and trafficking.

Previous studies have shown that both co-stimulation and cytokines are critical for T-cell differentiation as T cells that receive initial activation through MHC peptide interaction with the TCR and co-stimulation from various receptors without receiving a third signal from various cytokines undergo poor proliferation and express lower levels of effector molecules^47^, which is consistent with the activation profile of TCR_TAG_ cells delivered to untreated tumor-bearing mice. Our findings are also in line with the two-stage model of tumor-specific CD8^+^ T-cell activation wherein initial T-cell priming in tdLNs is followed by differentiation of tumor-infiltrating stem-like CD8^+^ T cells into effector cells within the tumor upon receiving additional co-stimulatory signals^47^. This also implies that antigen-specific T cells primed by DCs in the tdLNs must have access to their tumor targets and encounter favorable conditions in the perivascular TME for developing effector function. Thus, even in the context of successful T-cell priming, tumor lesions could be protected from immunological eradication by local immunosuppression and ECM crosslinking. For example, TCR_TAG_ cells entering the omental metastasis in untreated MOVCAR 5009-bearing mice might have received a strong TCR signal due to an abundance of tumor antigens in the perivascular niche. However, the inflammatory signals generated in the absence of ICD led to insufficient induction of inflammation-associated transcriptional factors or downstream inflammation-associated genes, resulting in weak antitumor responses^47, 54, 55^. Thus, the low co-stimulation in combination with negative regulatory signals provided by an influx of immunosuppressive myeloid cells as well as components of the fibrillar ECM could explain the reduced expansion and dysfunctional state of TCR_TAG_ cells. In contrast, the OV treatment-delivered ICD generated co-stimulatory signals in the perivascular TME that supported the differentiation of the transferred T cells. However, despite the same viral backbone in OV-Fc and OV-CXCR4-A, there were profound treatment-mediated differences regarding T-cell trafficking and effector program acquisition in the perivascular TME that were associated with antitumor efficacy. Such differences in antitumor responses elicited by these closely related ICD-inducing oncolytic viruses raised the question of criteria defining optimal levels of inflammation in the TME that could predict a successful outcome of immunotherapy. Particularly, the extent to which adjuvanticity of the OV-induced ICD can shape the T-cell effector phenotype in the perivascular TME through quantitative and qualitative changes in the infiltrating myeloid cell populations needs to be further interrogated. We hypothesize that OV-Fc treatment delivered before ACT induced strong inflammatory responses, leading to high activation of TCR_TAG_ cells that was counteracted by dysfunction, in part, due to an influx of suppressive myeloid cells in the tumor. For example, among antigens that were expressed in myeloid cells after OV-Fc but suppressed after OV-CXCR4-A treatment was HIF1a. Known for its multifactorial tumor-promoting effects, including angiogenesis, hypoxia, upregulation of several immune checkpoint proteins as well as the induction of Arg1/2 and nitric oxide in MDSCs^56, 57, 58^, the high expression level of HIF1a could contribute to the dysfunction of TCR_TAG_ cells and impair their trafficking. Independently of T-cell activation levels, the increased expression of some Lox family enzymes involved in ECM assembly^59^ could also limit the migration of TCR_TAG_ cells when delivered after OV-Fc treatment. In contrast to OV-Fc, and aligned with the ability of the virally-delivered CXCR4 antagonist to reduce the immunosuppressive network in the TME^14, 17, 18^, secretion of CXCR4-A into the perivascular tumor areas from virally infected tumor cells created milder inflammation that modulated the level and quality of myeloid cell responses associated with the generation of functional TCR_TAG_ cells.

The critical role of the perivascular TME during effector program acquisition by tumor-infiltrating TCR_TAG_ cells was further emphasized by a comparable profile of antigen priming in the tdLNs, supporting the notion that the signals driving the differentiation of tumorigenic T cells to an effector state occur in the tumor sites^47^. The analyzed tdLNs contained high numbers of antigen-experienced T cells with a sizable fraction of TCR_TAG_ cells exhibiting a stem-like phenotype^47, 60^, implicating their potential to undergo effector differentiation after migration to the tumor and receiving additional co-stimulation within the perivascular niche. This also implies that a controlled level of adjuvanticity associated with the mechanisms of cancer cell death is required to generate a permissive TME for the optimal effector phase of tumor-specific immune responses. It also delineates some molecular and cellular components of effector T-cell differentiation generated during OV-induced ICD with perivascular TME parameters standing out as critical determinants of regulated cell death immunogenicity. However, despite some shortcomings, ICD appears as an important treatment mode for cancer immunotherapy, particularly when used in combination with other modalities that have a major role in the control of OC and other neoplasms. The demonstrated antitumor synergy between OV-CXCR4-A and the adoptively transferred tumoricidal T cells complements our previous study wherein the same oncolytic virus in combination with doxorubicin increased ICD of drug-resistant OC cells concomitant with reversing the immunosuppressive TME and controlling metastatic growth in syngeneic mice^14^. In line with these and other preclinical findings, several ICD inducers were reported to positively interact with numerous immunotherapeutic approaches in patients with cancer, including anti-HER2 anthracycline-based antibody conjugate that potentiated PD-1 blockade in breast cancer^61^ among other malignancies^62, 63^. Several strategies have also been focused on overcoming ICD deficiencies such as intratumoral injection of pattern recognition receptor (PRR) agonists or recombinant type I IFNs^64, 65, 66^ or the inhibition of endogenous suppressors of adaptive immunity elicited by ICD, including CD47 blocking with specific monoclonal antibodies^67^. Altogether, these observations identify multiple strategies to increase the immunogenicity of clinically “cold” tumor variants by combining ICD inducers with other treatment modalities in the clinical setting to achieve regimens with superior clinical efficacy.

## Methods

### Mice and cell lines

6-8-week-old female B6.Cg-Tg(TcraY1,TcrbY1)416Tev/J mice, expressing a rearranged TCR transgene specific for the *H2-D*^b^-restricted SV40 large tumor antigen (TAG)_206−215_ epitope I (SAINNYAQKL), were obtained from The Jackson Laboratory (Bar Harbor, ME, USA) and housed in the Comparative Oncology Shared Resource (COSR) at Roswell Park Comprehensive Cancer Center (RPCCC) (Buffalo, NY, USA). Six-week-old female C.B-Igh-1b-Icr-Tac-Prkdc SCID/Ros (SCID) mice were obtained from the COSR at RPCCC (Buffalo). All animal studies were performed in compliance with the guidelines established by the Institutional Animal Care and Use Committee (IACUC) under approved protocols. The TAG-expressing MOVCAR 5009 ovarian carcinoma cell line, transduced with a retroviral construct encoding the firefly luciferase gene (pWZL-Luc) for *in vivo* imaging^23^, was kindly provided by Dr. Denise Connolly (Fox Chase Cancer Center, Philadelphia, PA, USA). MOVCAR 5009 cells were cultured in DMEM (Corning, NY, USA) supplemented with 10% fetal bovine serum (FBS; Corning), 5 μg/ml gentamicin sulfate (Corning), and maintained at 37°C with 5% CO_2_. The MOVCAR 5009 cell line was authenticated at the American Type Culture Collection (ATCC; Manassas, VA, USA) using short tandem repeat profiling.

### Oncolytic vaccinia viruses (OVs)

The OVs used in the study were of the Western Reserve stain with disrupted thymidine kinase (TK) and vaccinia growth factor (VGF) genes for enhanced cancer cell specificity. The generation and characterization of the virus expressing the Fc portion of murine IgG2a (OV-Fc) and CXCR4 antagonist in the context of the Fc portion of murine IgG2a (OV-CXCR4-A) have been described previously^17^.

### *In vivo* studies

SCID mice (n = 5 per group) were injected i.p. with 5 × 10^6^ MOVCAR 5009 cells and treated i.p. with 5 × 10^7^ PFU of OV-Fc or OV-CXCR4-A 10 days after the tumor challenge, while untreated mice served as control. For the adoptive T cell transfer (ACT), spleens from naïve B6.Cg-Tg(TcraY1, TcrbY1)416Tev/J mice were collected, homogenized, and filtered through a 70 μm cell strainer into ammonium-chloride-potassium (ACK) lysis buffer (Quality Biological, Gaithersburg, MD, USA) to lyse erythrocytes. Cells were washed twice in cold RPMI 1640 media (Corning) and 5 × 10^6^ TCR_TAG_ T cells, separated using Pan T cell isolation kit II (Miltenyi Biotech, Gaithersburg, MD, USA), were injected i.v. to MOVCAR 5009-bearing SCID mice 3 days after oncolytic virotherapy treatment. Tumor growth was monitored by bioluminescence imaging and signals were determined by IVIS Spectrum In Vivo Imaging System (PerkinElmer, Waltham, MA, USA) after i.p. injection of 200 μl D-Luciferin (150 mg/kg body weight; Gold Biotechnology, St. Louis, MO, USA) following the manufacturer’s protocol. The values for average radiance (photons/sec/cm^2^/sr) in ROIs were determined in the Living Image 4.7.3 Software for IVIS Spectrum.

### Immunohistochemistry (IHC)

Immunohistochemical staining was done on the omental tumor sections harvested from MOVCAR 5009-bearing SCID mice 10 days after OV treatments. Briefly, samples were placed in 10% neutral buffered formalin (NBF) for 24 h, dehydrated, and embedded in paraffin. Formalin-fixed paraffin-embedded (FFPE) sections (4 μm thick) were stained using a rat anti-mouse antibody specific for CD31 (clone: SZ31, DAI-310; Dianova, Eching, Germany) at 1:35 dilution for 40 min, followed by incubation with rabbit anti-rat IgG (ab102248; Abcam, Cambridge, UK) for 30 min, and Rabbit EnVision^+^ (K4003; Agilent Technologies, Santa Clara, CA, USA) for 30 min. Diaminobenzidine (DS9800; Leica Biosystems, Wetzlar, Germany) was applied for 10 min and slides were counterstained with hematoxylin for 8 min. Slides were scanned by the Aperio AT2 Slide scanning system and data were analyzed with the Aperio ImageScope 12.3.3 Software (Leica Biosystems). MVD was evaluated by enumerating the number of CD31^+^ endothelial clusters within a region of interest and microvessel diameter was measured at the vessel’s largest width as described^4, 17^.

### The multispectral immunofluorescence (mIF) imaging

#### Sample preparation and staining:

The mIF staining on FFPE omental tumor sections was performed by The Advanced Tissue Imaging Shared Resource (ATISR) at RPCCC using the Opal 6-Plex Detection Kit (NEL821001KT, AKOYA Biosciences, Marlborough, MA, USA) as described^68^. Briefly, FFPE 4 μm sections were cut and placed on charged slides. Slides were dried at 65°C for 2 h. After drying, the slides were placed on the BOND RXm Research Stainer (Leica Biosystems) and deparaffinized with BOND Dewax solution (AR9222, Lecia Biosystems). The mIF staining process involved serial repetitions of the following for each biomarker: epitope retrieval/stripping with ER1 (citrate buffer pH 6, AR996, Leica Biosystems) or ER2 (Tris-EDTA buffer pH9, AR9640, Leica Biosystems), blocking buffer (AKOYA Biosciences), primary antibody, Opal Polymer HRP secondary antibody (AKOYA Biosciences), Opal Fluorophore (AKOYA Biosciences). Spectral DAPI (AKOYA Biosciences) was applied once slides were removed from the BOND. They were cover-slipped using an aqueous method and Diamond antifade mounting medium (Invitrogen ThermoFisher Scientific, Waltham, MA, USA). The mIF panels consisted of the antibodies as detailed in Supplementary Table 1.

#### Tissue imaging and analysis:

Slides were imaged on the PhenoImager^™^ HT (AKOYA Biosciences). Further analysis of the slides was performed using inForm^®^ Software v2.6.0 (AKOYA Biosciences). The whole slides were first scanned in an unmixed view, then representative ROIs were selected for acquisition under the guidance of a pathologist. These ROIs were then rescanned to achieve full spectral unmixing. A representative subset of these unmixed ROIs was then used to train tissue and cell segmentation. Next, a unique algorithm was created using a machine-learning technique, in which the operator selects positive and negative cell examples for each marker. These algorithms were then batch-applied across a greater number of ROIs selected for inclusion in further analysis. The RStudio plugin, phenoptrReports, was used to extract phenotype counts from the resulting data tables.

### Spatial Transcriptomics

#### Sample preparation:

ST analysis was completed on FFPE sections prepared from omental tumors of MOVCAR 5009-bearing SCID mice 10 days after OV treatments using the 10x Genomics Visium platform by the Genomics Shared Resource (GSR) at RPCCC. After tissue mounting on Visium slides, hematoxylin and eosin (H&E) images were captured for downstream data analysis. FFPE blocks were stained with H&E, imaged for pathological review, sectioned at 5 μm, and trimmed to fit in the capture area (6.5 mm × 6.5 mm) of the Visium Spatial slides (10x Genomics, Pleasanton, CA, USA). Each area contains an array of ~ 5000 spots (55 μm in diameter). The tissue areas of interest were identified by a pathologist based on histologically well-organized CD31-expressing tumor vasculature. The RNAs within the tissue were hybridized to the whole transcriptome probe panel and the hybridized probes were captured on the Visium slides. Captured probe products were then extended using the unique molecular identifier (UMI), Spatial Barcode, and partial Read 1, and the obtained cDNA was used for gene expression library construction. Gene expression libraries for each sample were produced with enzymatic fragmentation, end-repair, a-tailing, adapter ligation, and PCR to add Illumina-compatible sequencing adapters. The resulting libraries were evaluated using D1000 Screen Tape on the TapeStation 4200 (Agilent Technologies) and quantitated using the KAPA Biosystems qPCR quantitation kit for Illumina. They were then pooled, denatured, and diluted to 300 pM (picomolar) with 1% PhiX control library added. The resulting pool was then loaded into the appropriate NovaSeq Reagent cartridge followed by sequencing on a NovaSeq6000 according to the manufacturer’s protocol (Illumina Inc., San Diego, CA, USA). Once sequencing was completed, tissue images taken after H&E staining on the Visium slide were used to align the gene expression from the spatial barcodes unique to each location in the capture area during data analysis.

#### 10x Genomics Visium data analysis:

For the 10x Genomics Visium analysis, mapping results (binary alignment and map [BAM] files), and quantification matrices were generated using Space Ranger v.1.3.1 software with the mouse mm10 genome and GENCODE annotation database. Then the filtered gene-barcode matrices, which contain barcodes with the UMI counts that passed the cell detection algorithm, were used for further analysis. All downstream analyses were performed using the Seurat single-cell data analysis R package. The normalized and scaled UMI counts were calculated using the SCTransform method. Differentially expressed genes between clusters and samples were identified using the FindMarkers function with the Wilcoxon rank-sum test from Seurat. Pathway analysis was carried out using the fgsea R package with the gene list ranked by average log2 fold change. The Hallmark (H) and the Canonical pathways (CP) of curated gene sets (C2) of the MSigDB pathway database were used in the pathway analysis. Activation/effector and dysfunction scores were calculated using the AddModueScore method from Seurat with selected genes in each functional category.

### Single Cell RNA sequencing

#### Sample preparation:

The scRNAseq analysis was performed on single-cell suspensions prepared from tumor-draining lymph nodes (tdLNs) of MOVCAR 5009-bearing SCID mice 10 days after oncolytic virotherapy treatments. The lymph nodes were harvested, homogenized, and obtained cells were resuspended in 0.04% BSA (Sigma-Aldrich, Burlington, MA, USA) in phosphate-buffered saline without calcium and magnesium (PBS; Corning), passed through a 70 μm cell strainer (Fisher Scientific, Waltham, MA, USA) to receive single-cell suspension and washed three times in pre-chilled 1% BSA in PBS. Single-cell gene expression libraries were created using the 10x Genomics Chromium Next GEM Single Cell 3’ Kit v.3.1 (10x Genomics). To evaluate the viability and number of cells, as well as the absence of clumps and debris in single-cell suspensions, trypan blue and a Countess FL automated cell counter (Thermo Fisher Scientific) were used. Subsequently, samples from different experimental groups (ACT, OV-Fc/ACT, OV-CXCR4-A/ACT) were loaded separately in equal amounts into the Chromium Controller (10x Genomics), and reverse transcription and cDNA amplification were performed. This full-length amplified cDNA was then used to generate transcriptome libraries by enzymatic fragmentation, end-repair, A-tailing, adapter ligation, and PCR to add Illumina-compatible sequencing adapters. Evaluation of the obtained libraries was achieved on D1000 screen tape using a TapeStation 4200 (Agilent Technologies) and quantitation using the Kapa Biosystems qPCR Quantitation Kit for Illumina. The libraries were denatured, diluted to 300 pM with 1% PhiX control library, loaded into the NovaSeq reagent cartridge, and sequenced on a NovaSeq6000 according to the manufacturer’s protocol (Illumina). The received sequencing data from the 10x Genomics libraries were processed in the Cellranger v.7.0.0 Software (10x Genomics).

#### scRNAseq analysis:

For the scRNAseq analysis, mapping results (binary alignment and map [BAM] files), and quantification matrices were generated using Cell Ranger v.7.0.0 Software with the mouse mm10 genome and GENCODE annotation database. Then the filtered gene-barcode matrices, which contain barcodes with the UMI counts that passed the cell detection algorithm, were used for further analysis. All downstream analyses were performed using the Seurat single-cell data analysis R package. First, cells with very low or high RNA feature content (< 500 or > 7500 genes detected) or higher mitochondrial RNA content (> 15%) were filtered out from the analysis to remove empty cells and doublets. Then, the normalized and scaled UMI counts were calculated using the SCTransform method. Subsequently, dimension reductions, including principal component analysis (PCA), UMAP, and t-distributed stochastic neighbor embedding (tSNE), were carried out using the highly variable genes. Cell clusters were identified using the shared nearest neighbor (SNN)-based clustering on the first 12 principal components. The cell clusters were annotated by SingleR packages using the ImmGen reference database of the cell dex R package. Differentially expressed genes between clusters and samples were identified using the FindMarkers function with the Wilcoxon rank-sum test from Seurat.

### Flow Cytometry

Flow cytometry analysis was performed on single-cell suspensions prepared from the inguinal tumor-draining lymph nodes of MOVCAR 5009-bearing SCID mice 10 days after oncolytic virotherapy treatment. Briefly, cells were incubated with an anti-mouse CD16/CD32 Fc blocking antibody (BD Biosciences, Franklin Lakes, NJ, USA) for 20 min at 4°C followed by extracellular staining with anti-mouse fluorochrome-conjugated antibodies for 30 min at 4°C in the dark. All antibodies were purchased from BD Biosciences or BioLegend (San Diego, CA, USA), as detailed in Supplementary Table 2. For the exclusion of dead cells, Live/Dead Fixable Aqua Stain was used according to the manufacturer’s instructions (Thermo Fisher Scientific). Samples were acquired with the LSR Fortessa flow cytometer (BD Biosciences) and FACSDiva Acquisition Software (BD Biosciences). Data analysis was performed using WinList 3D 9.0.1 (Verity Software House, Topsham, ME, USA).

#### Statistical analysis

Statistical analyses were performed using GraphPad Prism 10 (GraphPad Software Inc., San Diego, CA, USA) and R Software. Two-way ANOVA with Tukey’s multiple comparisons was used to determine significant differences in tumor growth between groups. The Wilcoxon rank sum test was used for testing differences between samples for mIF, 10x Genomics Visium, and single-cell RNA sequencing data. Fisher exact test was used for testing cell composition between samples. The Pearson correlation between activation/effector score and dysfunction score was tested using the stat cor method from the ggpubr package. Data are presented as mean ± S.D. For the box plots, the line inside the box shows the median, the lower and upper hinges correspond to the first and third quartiles (the 25th and 75th percentiles). The upper whisker extends from the hinge to the largest value no further than 1.5 * IQR from the hinge (where IQR is the inter-quartile range or distance between the first and third quartiles). The lower whisker extends from the hinge to the smallest value at most 1.5 * IQR of the hinge. Data beyond the end of the whiskers, “outlying” points, are plotted individually. The threshold for statistical significance was set to *p* < 0.05.

## Data Availability

The raw data of scRNA sequencing and Spatial Transcriptomics have been deposited in the database of Gene Expression Omnibus (GEO) under the accession number GSE274508 and GSE274509, respectively. The data supporting the findings reported in this study are available within the manuscript and supplementary information. Source data are provided in the Source Data file.
